# The Paracaspase MALT1 in Cancer

**DOI:** 10.3390/biomedicines10020344

**Published:** 2022-02-01

**Authors:** Beatriz Gomez Solsona, Anja Schmitt, Klaus Schulze-Osthoff, Stephan Hailfinger

**Affiliations:** 1 Interfaculty Institute of Biochemistry, University of Tuebingen, 72076 Tuebingen, Germany; beatriz.gomez-solsona@uni-tuebingen.de (B.G.S.); kso@uni-tuebingen.de (K.S.-O.); 2 Department of Medicine A, Hematology, Oncology and Pneumology, University Hospital Muenster, 48149 Muenster, Germany; anja.schmitt@ifib.uni-tuebingen.de; 3 German Cancer Consortium (DKTK) and German Cancer Research Center (DKFZ), 69120 Heidelberg, Germany; 4 Cluster of Excellence iFIT (EXC 2180) “Image-Guided and Functionally Instructed Tumor Therapies”, University of Tuebingen, 72076 Tuebingen, Germany

**Keywords:** MALT1, BCL10, CARD11, CARD10, CBM complex, NF-κB, paracaspase, protease

## Abstract

Almost twenty years ago, the importance of the paracaspase MALT1 in antigen receptor-induced NF-κB activation was first described. Since then, several other immune receptors, G-protein-coupled receptors, and receptor tyrosine kinases were identified as relying on MALT1 to induce NF-κB activation. In various hematological malignancies and solid tumors, MALT1 is constitutively activated and drives chronic NF-κB target gene expression. Deregulated MALT1 activity in cancer thus promotes tumor cell survival, proliferation, and metastasis. Since the molecular function of MALT1 partially requires its protease activity, pharmacological targeting of MALT1 may represent a promising anti-cancer strategy. Here, we review the molecular features of MALT1 activation and function as well as the therapeutic potential of MALT1 inhibition in hematological malignancies and solid tumors.

## 1. Introduction

The transcription factor NF-κB promotes cancer development on various levels, e.g., by enhancing cell survival and proliferation, the acquisition of cancer stem cell properties, metastasis formation, metabolic reprogramming, and the suppression of anti-tumor immune responses [1,2]. Thus, targeting the activity of NF-κB has emerged as a promising anti-cancer strategy. However, a major setback of this therapeutic approach has become evident in mouse models lacking the expression of central regulators that are important for the general activation of the classical NF-κB pathway. Mice deficient in signaling components, such as the inhibitor of κB kinase (IKK) complex, are not viable and exhibit increased hepatocyte apoptosis, immune deregulation, as well as skeletal and epidermal defects, indicating an essential role of NF-κB in tissue homeostasis [3]. Accordingly, clinical trials of several IKK inhibitors have been discontinued due to unfavorable safety profiles [4]. One possibility to circumvent the complications of a general NF-κB inhibition is to focus on specific upstream regulators that can induce NF-κB activation but are not essential for the survival of the organism. One of these NF-κB regulators is the mucosa-associated lymphoid tissue lymphoma translocation protein 1 (MALT1) which has recently emerged as an interesting therapeutic target. Here, we will summarize what is known about the molecular function of MALT1 and how it is activated in hematological malignancies and solid cancers, and discuss the therapeutic potential of MALT1 inhibitors as well as their impact on immunotherapy.

## 2. MALT1 Is Activated by CARD-CC Proteins

**Figure 1:**
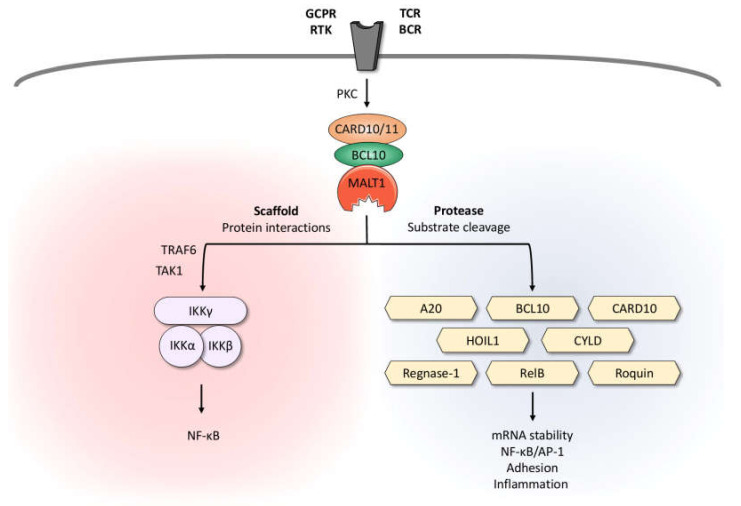
Dual role of MALT1: Scaffold and protease function. Upon triggering of antigen receptors, various GPCRs and RTKs, MALT1 is activated in the context of a high-molecular-weight complex comprising BCL10 and CARD10 in non-hematopoietic or CARD11 in hematopoietic cells, respectively. By recruiting the IKK complex, MALT1 serves as a scaffold to promote optimal activation of the transcription factor NF-κB. Due to its protease activity, MALT1 cleaves various substrates involved in mRNA stability, regulation of NF-κB/AP-1 signaling, adhesion, and inflammation. BCL10, B-cell lymphoma/leukemia 10; BCR, B-cell receptor; CARD10/11, caspase recruitment domain-containing protein 10/11; GPCR, G-protein-coupled receptor; HOIL1, heme-oxidized IRP2 ubiquitin ligase 1; IKK, inhibitor of κB kinase; MALT1, mucosa-associated lymphoid tissue lymphoma translocation protein 1; PKC, protein kinase C; RTK, receptor tyrosine kinase; TAK1, transforming growth factor-β-activated kinase 1; TCR, T-cell receptor; TRAF6, TNF receptor associated factor 6.

Proteins that contain a caspase activation and recruitment domain (CARD) and a coiled-coil (CC) domain are critical for the activation of MALT1. So far, four CARD-CC proteins, which differ in their tissue expression profiles, have been described. CARD9 is mainly expressed in cells of the myeloid lineage, such as macrophages or neutrophils, and links multiple innate immune receptors, such as TREM1 or Dectin-1, to NF-κB activation [5,6,7]. Lymphocytes express the CARD-CC member CARD11 (also known as CARMA1), which transmits signals from the B-cell, T-cell, or activating Natural Killer cell receptors to MALT1 [5,8,9,10]. In contrast, the expression of CARD10 and CARD14 is mainly restricted to non-hematopoietic tissues. Whereas CARD14 (also known as CARMA2) is predominantly expressed in keratinocytes and involved in the NF-κB activation downstream of pattern recognition receptors (PRR), such as Dectin-1, CARD10 (also known as CARMA3) is expressed in several tissues (e.g., intestine, heart, and kidney) and connects some G-protein-coupled receptors (GPCRs) and receptor tyrosine kinases (RTKs) to MALT1 and NF-κB activation [5,11,12,13,14,15,16]. Even though the molecular events involved in T-cell receptor-mediated CARD11 activation have been elucidated in great detail, it is not completely understood how other immune receptors, RTKs, and GPCRs promote the activation of the different CARD-CC proteins. However, it has been proposed that, due to the homology of the CARD-CC members, major steps in their activation might be conserved [5,17]. An intramolecular interaction between the CARD, the CC domain, and a flexible linker region keeps the CARD-CC proteins in an inactive state [18,19]. This auto-inhibitory conformation is disrupted by a protein kinase C (PKC)-mediated phosphorylation of the linker region, which induces a structural constraint on this flexible domain of CARD11 [20,21]. The open conformation of phosphorylated CARD11 then allows oligomerization driven by the coiled-coil domain and the CARD-mediated recruitment of B-cell lymphoma 10 (BCL10) [5,8,22,23]. Oligomerized CARD11 serves as a platform that allows the polymerization of BCL10, leading to the formation of helical BCL10 filaments [24,25]. As BCL10 constitutively interacts with MALT1, its recruitment also translocates MALT1 to activated CARD11 oligomers and brings individual MALT1 molecules into close proximity. MALT1 possesses two distinct molecular functions promoting the activation of NF-κB. On the one hand, its protease activity acquired in the context of the CARD-BCL10-MALT1 (CBM) complex is important for the optimal activation of NF-κB (please see the following chapter). On the other hand, together with BCL10 it serves as a scaffold to recruit E3 ubiquitin ligases, such as TRAF6 and the linear ubiquitin chain assembly complex (LUBAC), resulting in K63- and M1-linked ubiquitination of BCL10 and MALT1 [26,27,28,29] (Figure 1). These polyubiquitin chains serve as docking sites for two distinct ubiquitin-binding complexes: The IKK complex is recruited via the ubiquitin-binding domain of the subunit IKKγ (also known as NEMO), whereas the transforming-growth-factor-β-activated kinase 1 (TAK1) associates with polyubiquitin chains via the adapter TAK-binding protein 2 (TAB2) [26,27,29,30]. Through ubiquitination of IKKγ and TAK1-mediated phosphorylation of IKKβ, the IKK complex becomes activated and subsequently phosphorylates the inhibitor of κB (IκB), causing its proteasomal degradation [31,32,33]. This allows the transient nuclear translocation of NF-κB members that in turn drive target gene transcription. Taken together, various receptors, such as the antigen receptors or PRRs, promote the formation of a CBM complex, and thus the activation of classical NF-κB transcription factors.

## 3. MALT1 Protease Function

The central caspase-like domain of MALT1 has been bioinformatically identified due to its homology to the protease domains of caspases and it is responsible for the protease activity of MALT1 [34]. Whereas in non-activated T cells, TRAF6 and GRK2 suppress the protease activity of MALT1, monoubiquitination and dimerization activate MALT1 protease function, leading to the cleavage of selected substrates [35,36,37,38]. In contrast to caspases, which cleave their substrates after the acidic amino acid aspartate, MALT1 cleaves proteins after the positively charged arginine [39,40]. The MALT1 substrates identified so far can be divided into three groups. The first group comprises proteins that affect the activation of the transcription factors NF-κB and activator protein 1 (AP-1). MALT1-mediated inactivation of the deubiquitinating enzymes A20 and CYLD protects K63-linked polyubiquitin chains from degradation and thus sustains NF-κB and AP-1 activation [40,41]. The MALT1-dependent cleavage of the NF-κB member RelB leads to its rapid degradation by the proteasome, thus freeing the NF-κB subunits RelA and c-Rel from transcriptionally inactive complexes [42]. Interestingly, MALT1-mediated cleavage of the LUBAC member HOIL1 might represent a negative feedback loop that limits CBM-dependent NF-κB activation [43,44,45]. The second group of MALT1 substrates includes proteins that regulate the stability and the translation of selected transcripts. Regnase 1, roquin 1, and roquin 2 control a variety of inflammation-related mRNAs, such as NFKBIZ (coding for the atypical IκB member IκBζ), IL6, TNFA, ICOS, and TNFAIP3 (coding for A20). MALT1-mediated inactivation of regnase 1 and roquin 1/2 abrogates their capacity to suppress mRNA translation and thus promotes the expression of the immunoregulatory transcripts [46,47]. The third group of MALT1 substrates contains members of the CBM complex itself. MALT1 auto-proteolysis promotes NF-κB activity, even though the molecular basis of this observation is not yet fully understood [48]. In contrast, MALT1-mediated BCL10 cleavage is dispensable for NF-κB activation, but promotes T-cell receptor-induced cell adhesion to fibronectin [39].

Since MALT1 protease activity has been shown to control glutamine uptake by ASCT2 and glutaminolysis in various cell types, MALT1 also appears to be a key regulator of glutamine metabolism, suggesting that there are so far uncharacterized MALT1 substrate(s) [49,50,51].

## 4. MALT1 in Hematological Malignancies

Since numerous reviews have highlighted the role of MALT1 in hematological malignancies, this review mainly focuses on the importance of MALT1 in solid tumors and only briefly summarizes its role in lymphoma and leukemia development [52,53,54]. As its name implies, MALT1 was first described in mucosa-associated lymphoid tissue (MALT) lymphoma, in which the *MALT1* gene is often subject to translocations and thus was suspected to be a driver of lymphomagenesis [55]. At present, it is broadly accepted that constitutive activation of MALT1 by either genetic alterations, chronic antigen receptor signaling, overexpression, or viral proteins drives the development of several hematological malignancies.

MALT1 overexpression has been reported mainly in MALT lymphoma, but it can also be observed in other malignancies, such as B-cell acute lymphoblastic leukemia (B-ALL) and diffuse large B-cell lymphoma (DLBCL) [56,57,58,59,60]. Overexpression of MALT1 is caused either by amplification of its gene locus or by the translocation t(14;18)(q32;q21), which brings the *MALT1* gene under the control of the IgG heavy chain promoter [57,59]. The oncogenic potential of MALT1 has been demonstrated in a mouse model that allows specific MALT1 expression in hematopoietic stem/progenitor cells which results in the development of MALT lymphoma [61]. Additional deletion of the tumor suppressor p53 accelerated tumor onset and resulted in an aggressive form of DLBCL, highlighting MALT1′s potential to promote B-cell survival and proliferation [61]. Similarly, the overexpression of BCL10, which induces MALT1 activation, also provokes lymphomagenesis in mice [62]. Mutations of CARD11, which can be found in an aggressive subtype of DLBCL, Sézary syndrome, angioimmunoblastic T-cell lymphoma (AITL), and adult T-cell leukemia/lymphoma (ATLL), disrupt the auto-inhibition of CARD11 and drive its receptor-independent oligomerization, thus activating MALT1 [63,64,65,66].

One third of MALT lymphomas harbor a translocation (i.e., t(11;18)(q21;q21)) which fuses the N-terminal part of cellular inhibitor of apoptosis protein 2 (cIAP2, also known as API2) to the C-terminus of MALT1 [55,67]. The cIAP2-MALT1 fusion protein is able to oligomerize independently of BCL10 and upstream signals and, through TRAF6 recruitment and MALT1 protease activation, induce NF-κB-dependent transcription [40,68,69]. Strikingly, due to cIAP2-mediated recruitment, two non-canonical MALT1 substrates are processed by the cIAP2-MALT1 fusion protein, i.e., the NF-κB-inducing kinase (NIK) and the LIM domain and actin-binding protein 1 (LIMA1). NIK cleavage results in an increased stability of its C-terminal fragment and in the activation of noncanonical NF-κB signaling, whereas LIMA1 processing converts the tumor suppressor into an oncogene [70,71].

Besides genetic alterations of components of the CBM signalosome, constitutive antigen receptor signaling is a major driver for chronic MALT1 activation and thus for lymphoma development. Chronic active B-cell receptor signaling is caused by several distinct molecular mechanisms. In DLBCL, self-antigens that are either present on the BCR itself or in apoptotic debris of the tumor cells have been proposed to promote the activation of the lymphoma cells [72]. In chronic lymphocytic leukemia (CLL), CBM activation can be driven by BCR-derived signals that are antigen-independent but rely on the recognition of an internal epitope within the BCR [73]. Moreover, antigens deriving from persistent chronic infections with viral or bacterial pathogens, such as the hepatitis C virus, can promote lymphoma development by sustained B-cell activation and proliferation [74,75].

Interestingly, some viral proteins are also able to promote the activation of NF-κB via MALT1 in a BCR-independent manner. Latent infections with Kaposi’s sarcoma-associated herpes virus (KSHV) are correlated with the development of primary effusion lymphoma (PEL). The KSHV proteins K13 and K15 promote MALT1 activity and, therefore, regulate viral latency and the growth of PEL cells [76].

In other lymphomas, such as mantle cell lymphoma (MCL), chronic activation of BCR signaling and MALT1 activity have been detected, but the molecular cause currently remains elusive [77]. Even though MALT1 exhibits two distinct molecular functions that lead either to IKK activation (scaffold function) or to the cleavage of selected substrates (protease function), the inhibition of MALT1 protease activity is sufficient to reduce tumor cell proliferation and apoptosis resistance in DLBCL, MCL, PEL, ATLL, MALT, and T-cell acute lymphoblastic leukemia (T-ALL), highlighting the therapeutic potential of MALT1 inhibitors in these hematological malignancies [70,76,77,78,79,80,81].

## 5. The Role of the CBM Complex in Solid Tumors

Whereas the importance of the CBM signalosome in the pathogenesis of lymphoid malignancies has been acknowledged for years, recent studies now highlight CARD10-mediated MALT1 activation in solid tumors.

Amongst the tumor types in which NF-κB is activated through the CARD10-BCL10-MALT1 (C10BM) signalosome to promote tumorigenesis, there is a subset of angiotensin II receptor 1 (AGTR1)-positive luminal breast cancers with poor prognosis [82]. A recent study has provided evidence to support the existence of an AGTR1-C10BM-NF-κB signaling axis driving breast cancer (BC) cell-intrinsic and extrinsic responses. Gene expression dataset analysis has revealed a strong upregulation of the GPCR family member AGTR1 that correlates with an NF-κB gene signature in a subset of luminal breast cancers. Even though AGTR1 overexpression alone triggers considerable NF-κB activation, as shown by RelA nuclear translocation, the presence of angiotensin II (AGTR1′s ligand) further enhances NF-κB activity more than 100-fold and leads to the development of an aggressive phenotype. Interestingly, silencing of any constituent of the C10BM complex completely abrogates the angiotensin II-induced NF-κB response and thus blocks cell-intrinsic proliferative and invasive properties, as well as cell-extrinsic effects, such as the stimulation of endothelial cells of the tumor microenvironment to promote tumor angiogenesis [82].

Similarly, in proteinase-activated receptor 1 (PAR1)-driven tumors, both inhibition and silencing of MALT1 impairs the expression of NF-κB target genes, such as *IL1B*, *CXCL8*, and *MMP9*, each associated with the manifestation of malignant features in breast cancer and osteosarcoma (OS) [83]. Accordingly, the number and size of metastatic tumor nodules found in the lungs of nude mice injected with MALT1-deficient MCF7-N55 breast cancer cells are greatly attenuated compared to controls. These results highlight the role of the C10BM signalosome in the acquisition of pro-tumorigenic traits in PAR-1-dependent tumor entities through activation of NF-κB. Additionally, a recent study has underlined the role of MALT1 downstream of AGTR1 and PAR1 in the induction of triple-negative breast cancer (TNBC) subtype switching [84]. Typical estrogen receptor (ER)-positive luminal breast cancers that overexpress either AGTR1 or PAR1 exhibit NF-κB-driven epithelial-mesenchymal transition (EMT)-associated alterations, such as increased expression of Snail, ZEB1, vimentin, and N-cadherin, downregulation of E-cadherin, as well as increased cell invasion and migration—all of which are abrogated upon silencing or pharmacological inhibition of MALT1 [84]. Altogether, this reveals an important role of the MALT1 protease in the induction of the EMT program in GPCR-positive TNBCs.

In the context of a GPCR-activated C10BM signalosome, a study in ovarian cancer (OC) cells has uncovered a lysophosphatidic acid (LPA) receptor-PKCα-C10BM signaling axis that induces tumor progression via activation of NF-κB and urokinase plasminogen activator (uPA) [85]. In brief, lysophosphatidic acid (LPA) binds the GPCR LPAR1, which results in Ras activation. Ras subsequently activates the PKCα isoform, thus inducing the assembly of the C10BM signalosome and MALT1 activation. This PKC-C10BM signaling axis seems to be essential for NF-κB-dependent pro-tumorigenic features, such as invasion and migration, since silencing or inhibition of MALT1 completely abrogates NF-κB activation, uPA upregulation, and the appearance of malignant traits [85].

As mentioned above, C10BM complex assembly can also occur downstream of RTKs. Along this line, epidermal growth factor receptor (EGFR), a member of the RTK family, is aberrantly activated in various aggressive tumors including glioblastoma multiforme (GBM) and lung cancer. In these tumor entities, deregulation of the EGFR-NF-κB signaling pathway contributes to several important malignant properties of the tumor cells. Recent studies have now identified the C10BM complex as the central signaling hub through which EGFR ligation induces NF-κB activation. In this regard, Liu et al. have reported that EGF-dependent IκBα degradation and subsequent nuclear translocation of NF-κB are dependent on the expression of MALT1 [86]. Thus, MALT1 appears to be crucial for the maintenance of the proliferative and clonogenic potential of various GBM cell lines, and its silencing or pharmacological inhibition significantly reduces the invasive and migratory potential of the tumor cells both in vitro and in vivo [86]. Consistent with these results, a study by Pan et al. further supports a role for MALT1 in EGFR-mediated NF-κB activation [87]. The authors have demonstrated that the silencing of either CARD10 or MALT1 in EGFR-overexpressing lung cancer cells correlates with reduced NF-κB activity, cancer cell proliferation, migration, survival, and tumor burden in vivo. Intriguingly, contrary to what has been observed in the GBM model, the protease activity of MALT1 is largely dispensable for EGFR-induced NF-κB activation in lung carcinoma, since the reconstitution of MALT1-silenced cells with a protease-deficient mutant MALT1 (MALT1^C464A^) fully restores NF-κB signaling. Furthermore, knockdown of the MALT1 interaction partner TRAF6 abolishes NF-κB activation, pinpointing the importance of MALT1 as a scaffold protein to bridge TRAF6 to the IKK complex in response to EGF in this tumor entity. Interestingly, this study has also provided genetic evidence indicating that STAT3 activation in lung carcinoma is controlled by EGFR-MALT1-dependent NF-κB activation via induction of IL-6 expression.

Another example of an RTK that activates NF-κB via the C10BM signalosome is the human epidermal growth factor receptor 2 (Her2) protein, a member of the EGF receptor subclass that is frequently overexpressed in breast cancer [88]. Silencing of either one of the CBM complex members in Her2-overexpressing cells significantly reduces Her2-induced NF-κB activation. To determine the relationship between Her2 expression and C10BM activation, Pan et al. treated SKBR3 breast cancer cells with a PKC inhibitor. Interestingly, PKC inhibition is sufficient to completely abolish NF-κB activation, suggesting PKC as the functional link between Her2 overexpression and C10BM-induced NF-κB signaling. As observed for other tumor entities, the C10BM-NF-κB axis is responsible for the induction of tumorigenic properties in SKBR3 breast cancer cells, including proliferation, clonogenic survival, invasion, and migration [88]. In this context, invasion and migration have been shown to be promoted by the NF-κB-dependent expression of the matrix-remodeling enzymes MMP1 and 13. Consistent with what has been observed in other studies, injection of *CARD10*-silenced SKBR3 cells into nude mice leads to the formation of significantly smaller tumors and a reduced number of lung metastatic sites [88]. Furthermore, several other studies have identified MALT1 as a central regulator of NF-κB-mediated cancer progression. Both pancreatic ductal adenocarcinoma (PDAC) and prostate cancer (PCa) cells show increased MALT1 expression that contributes to tumor malignancy and metastasis [89,90]. Silencing or pharmacological inhibition of MALT1 reduces the proliferative and invasive potential of the tumor cells both in vitro and in vivo.

In malignant melanoma (MM) and cholangiocarcinoma (CCA), MALT1 has been reported to be overexpressed and thus to contribute to survival and metastasis [91,92]. A study by Wang et al., on melanoma proposes that the elevated MALT1 levels increase the TNFα- and TRAIL-induced NF-κB and JNK/AP-1 activation, thus promoting melanoma growth and pulmonary metastasis in vivo [91]. Interestingly, using the multi-kinase inhibitor regorafenib in intrahepatic CCA, the Raf/Erk/Elk-1 pathway has been found to modulate MALT1 expression [92]. The transcription factor Elk-1 directly binds to the *MALT1* promoter, thus contributing to MALT1 overexpression in the tumor cells. Accordingly, regorafenib treatment of CCA cells not only decreases MALT1 levels, but also reduces the expression of NF-κB target genes, such as *IL1B* and *CXCL8*, which correlate with CCA cell growth and survival [92].

Although the expression and activity of MALT1 and NF-κB are tightly controlled, some tumor cells can override these negative regulatory mechanisms to favor tumor progression. For instance, glioblastomas can transition from the more benign proneural (PN) subtype to the more aggressive mesenchymal (MES) subtype by downregulating the expression of the microRNA (miRNA) miR-181d [93]. It has been observed that in PN GBM, miR-181d can directly bind to the 3′-untranslated region (UTR) of *MALT1* transcripts, thereby reducing its mRNA and protein expression levels. This in turn results in a decrease in NF-κB transcriptional activity, as observed by the reduction in IL-6 secretion, as well as in a suppression of glioma cell proliferation, invasion, migration, and angiogenesis. The high-grade MES GBM can downregulate miR-181d, thus increasing MALT1-dependent NF-κB activation and enhancing tumor burden and aggressiveness [93]. Likewise, TIFA (TNF receptor associated factor-interacting protein with a forkhead-associated domain), which can sensitize hepatocellular carcinoma (HCC) cells to apoptosis, competes with MALT1 for TRAF6 binding [94]. A recent study has found that TIFA is downregulated in HCC cells, allowing free access of MALT1 to TRAF6, thus driving tumorigenesis through NF-κB activation.

Whereas most studies describe a tumor-promoting role for MALT1, a recent study suggests that MALT1 can also act as a tumor suppressor. In the lung cancer cell line A549, MALT1 cleaves the C10BM complex member CARD10 after the arginine residue at position 587, thereby dampening its capacity to activate NF-κB [95]. In this manner, the cleavage of CARD10 by MALT1 acts as a negative feedback mechanism that limits the signaling potential and pro-tumorigenic role of CARD10. This is also reflected in the observation that A549 knock-in (KI) cells expressing the cleavage-deficient R587A CARD10 variant exhibit a growth advantage, increased angiogenesis, and tumorigenic potential upon *i.v.* injection in mice [95]. The authors identified IL-6 as the main cytokine upregulated in the A549-KI cells, suggesting that cleavage of CARD10 by MALT1 is important to limit IL-6 signaling and to suppress tumorigenesis.

Collectively, these studies highlight the role of the C10BM signalosome as a critical signaling node for the pathogenic activation of NF-κB. MALT1 activity controls the expression of several cytokines and mediators that contribute to tumorigenesis by promoting proliferation, survival, invasion, migration, angiogenesis, and metastasis (Table 1). Thus, pharmacological targeting of MALT1 could represent a potential strategy in the treatment of solid tumors.

## 6. MALT1 as a Potential Therapeutic Target in Solid Tumors

### 6.1. Effects of MALT1 Inhibition on Tumor Cells

Constitutive MALT1 activity has been found to drive proliferation, survival, and metastasis in several tumor entities, such as lymphoid malignancies and solid tumors, and it is considered a crucial player in persistent NF-κB activation. The important role of the C10BM complex in the development of malignancy features in non-hematological tumors suggests a potential therapeutic benefit by targeting one of the complex members. Since MALT1 is the only constituent of the C10BM complex that exhibits enzymatic activity and since several protease inhibitors have already been developed, as recently summarized by Hamp et al., MALT1 represents the most suitable target in solid tumors [98]. Indeed, the use of either allosteric or active site MALT1 inhibitors has revealed significant anti-tumor effects in the treatment of solid tumors both in vitro and in vivo. For instance, treatment of metastatic melanoma, cholangiocarcinoma, and glioblastoma cells with the widely used active site MALT1 inhibitor MI-2 abrogates proliferation, migration, and invasion, reduces cell viability, and diminishes tumor growth and metastasis in vivo via downregulation of the NF-κB pathway [86,91,92]. Similarly, PAR1-driven metastasis-associated gene expression in breast cancer and osteosarcoma cell lines can be impaired by treatment with MI-2 and the suicide inhibitor z-VRPR-fmk [83]. Additionally, MI-2 abrogates PAR1-driven invasion of breast cancer cells [83]. However, in the context of melanoma, *MALT1* knockdown also suppresses activation of the JNK/AP-1 signaling pathway, indicating that the cellular effects observed upon MALT1 protease inhibition might not be attributable solely to the specific downregulation of NF-κB. Past studies have shown that in T cells, JNK phosphorylation depends on MALT1′s scaffolding function, indicating that the manifestation of some malignant traits might not be affected by targeting MALT1 protease function [39,99]. Allosteric MALT1 inhibitors, such as mepazine or thioridazine, have been used to treat TNBC and PDAC cells. The effects observed with these inhibitors include a reduction in the proliferative, invasive, and migratory capacities of the tumor cells, as well as an inhibition of primary tumor growth and metastatic burden in vivo. Conversely, the use of the active site inhibitor z-VRPR-fmk has no effect on lung carcinoma cells, since NF-κB activation and the development of tumorigenic features in this tumor type is regulated by MALT1′s scaffold function. In cases in which the protease function of MALT1 is dispensable, the development of inhibitors or biological entities that prevent either CARD10 oligomerization, C10BM complex formation, or TRAF6 recruitment would be inevitable [100].

### 6.2. Priming of Solid Tumors via MALT1 Inhibition

A promising new line of therapeutic strategy employing MALT1 inhibitors in solid tumors is based on their effect on regulatory T cells (Tregs). Mice expressing a mutant MALT1 that lacks protease activity exhibit a reduced frequency of natural regulatory T cells and suffer from severe autoimmunity [99,101,102]. Recent studies have demonstrated that the C11BM complex is important for the conversion of resting into effector Tregs, c-Myc-mediated Treg expansion and the immunosupressive effects of Tregs [103,104]. Moreover, conditional deletion of CARD11 exclusively in Foxp3-positive cells is fatal and associated with a multi-organ inflammatory disease [105]. Excitingly, incomplete inhibition of the C11BM complex either by reducing CARD11 expression or by pharmacological inhibition of MALT1 prevents lethal autoimmune reactions, but converts tumor-infiltrating Treg cells into IFNγ-secreting effector T cells [105]. This was sufficient to reduce the growth of the melanoma cell lines D4M.3A and B16F1 in mouse xenograft models [103,105]. Since the increased IFNγ production during incomplete C11BM inhibition provokes PD-L1 expression in the melanoma cells, the combination of MALT1 inhibitors and anti-PD1 immune checkpoint therapy exhibits synergistic effects in the inhibition of tumor growth [105]. Whether it is possible to achieve a MALT1 inhibition strong enough to boost the immune response by inhibiting Treg expansion and function, while avoiding severe systemic autoimmunity at the same time, is still under debate [106,107]. Nevertheless, it is tempting to speculate that a short-term application of MALT1 inhibitors in combination with immune checkpoint inhibitors could support the T cell-mediated anti-tumor reaction and thus prove beneficial for cancer patients.

**Figure 2:**
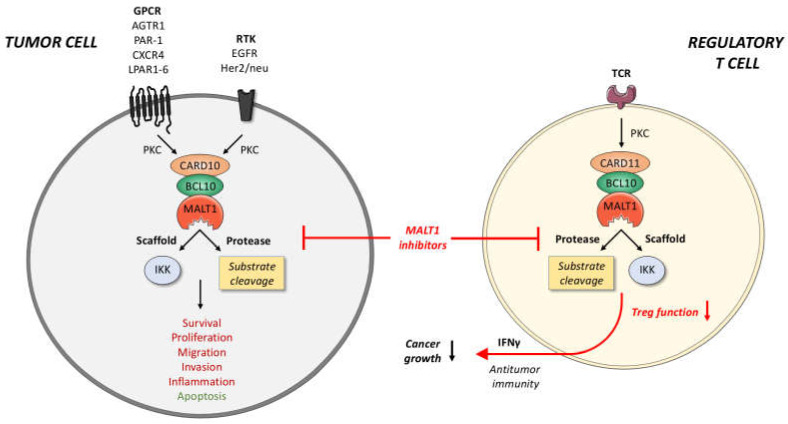
Potential anti-cancer effects of MALT1 inhibitors. In tumor cells, various GPCRs and RTKs can promote the assembly of a CARD10-BCL10-MALT1 complex. Through both its scaffold and protease function, MALT1 can activate transcription factors, such as NF-κB and AP-1, that mediate key processes in cancer cells, such as cell survival, proliferation, metastasis, and inflammation. In regulatory T cells, the CARD11-BCL10-MALT1 complex has been identified as crucial factors for Treg expansion and function. Pharmacological inhibition of MALT1 protease function not only interferes with the growth and aggressiveness of the tumor, but can also dampen and reprogram Treg activity, thus provoking a T cell-based anti-tumor immune response. Tumor-suppressive functions of MALT1 are highlighted in green, oncogenic effects in red. BCL10, B-cell lymphoma/leukemia10; CARD10/11, caspase recruitment domain-containing protein 10/11; GPCR, G-protein-coupled receptor; IKK, inhibitor of κB kinase; MALT1, mucosa-associated lymphoid tissue lymphoma translocation protein 1; PKC, protein kinase C; RTK, receptor tyrosine kinase; TCR, T-cell receptor.

Collectively, the inhibition of the MALT1 protease appears to represent a promising treatment strategy for solid tumors, if properly balanced. Potentially, MALT1 inhibition could be used to simultaneously target both tumor cells and suppressive immune cells (Figure 2). In CBM-dependent tumor cells, MALT1 inhibition directly acts on their proliferative and invasive potential, while, at the same time, it destabilizes the Treg-mediated immunosuppression. However, it is necessary to conduct further studies to adequately adjust the dosage and timing of these inhibitors for the treatment of solid tumors to prevent unwanted side effects, as well as to further evaluate possible drug combinations that could harness an improved anti-tumor effect.

## Figures and Tables

**Table 1:** Importance of MALT1 in different tumor types. AGTR1, angiotensin II receptor 1; ATL, adult T-cell lymphoma; B-ALL, B-cell acute lymphoblastic leukemia; BC, breast cancer; BCR, B-cell receptor; CARD11, caspase recruitment domain-containing protein 11; CCA, cholangiocarcinoma; CLL, chronic lymphocytic leukemia; DLBCL, diffuse large B-cell lymphoma; EGFR, epidermal growth factor receptor; GBM, glioblastoma; HER2, human epidermal growth factor receptor 2; HTLV-1; human T-cell lymphotropic virus type 1; KSHV, Karposi’s sarcoma-associated herpesvirus; LPAR, lysophosphatidic acid receptor; MALT1, mucosa-associated lymphoid tissue lymphoma translocation protein 1; MALT lymphoma; mucosa-associated lymphoid tissue lymphoma; MCL mantle cell lymphoma; MM, malignant melanoma; NSCLC, non-small cell lung cancer; OC, ovarian cancer; OS, osteosarcoma; PAR1, protease-activated receptor 1; PCa, prostate cancer; PDAC, pancreatic ductal adenocarcinoma; PEL, primary effusion lymphoma; T-ALL, T-cell acute lymphoblastic leukemia; TRAIL, tumor necrosis-factor-related apoptosis-inducing ligand.

**Hematological Malignancies**	**Role of MALT1**	**MALT1 Activation**	**Reference**
ATL	Proliferation Survival Anti-apoptotic signaling NF-κB target gene expression	HTLV-1 infection	[81]
B-ALL	Proliferation Survival Anti-apoptotic signaling	MALT1 overexpression	[56]
CLL	Cellular activation Proliferation Survival Anti-apoptotic signaling NF-κB target gene expression	Autonomous BCR-derived signaling	[80]
DLBCL	Proliferation Survival Anti-apoptotic signaling NF-κB target gene expression Pro-inflammatory signaling	Chronic BCR activation CARD11 mutations	[78,79]
MCL	Proliferation Survival Regulation of MYC expression	Chronic BCR activation	[77]
MALT lymphoma	Proliferation Survival	Chromosomal translocations resulting in MALT1 overexpression or fusion products	[70,96]
PEL	Survival	KSHV infection	[76]
T-ALL	Proliferation Survival Anti-apoptotic signaling NF-κB target gene expression	CARD11 overexpression	[97]
**Solid Tumors**	**Role of MALT1**	**MALT1 Activation**	**Reference**
BC	Proliferation Migration Invasion Gene reprogramming Angiogenesis NF-κB target gene expression EMT Metastasis	AGTR1 PAR1 HER2	[82,83,84,88]
CCA	Proliferation Growth Survival NF-κB target gene expression	Raf/Erk/Elk-1 pathway	[92]
GBM	Proliferation Clonogenicity Migration Invasion NF-κB target gene expression Metastasis	EGFR	[86]
NSCLC	Proliferation Survival Migration NF-κB target gene expression Metastasis	EGFR	[87]
MM	Proliferation Survival Growth NF-κB target gene expression Metastasis	TRAIL receptor	[91]
OS	Proliferation Survival Growth NF-κB target gene expression	PAR1	[83]
OC	Invasion Migration NF-κB target gene expression	LPA receptor	[85]
PDAC	Proliferation Invasion NF-κB target gene expression Metastasis	MALT1 overexpression	[89]
PCa	Proliferation Invasion NF-κB target gene expression Metastasis	MALT1 overexpression	[90]

## Data Availability

Not applicable.
